# Feasibility of Silver Doped TiO_2_/Glass Fiber Photocatalyst under Visible Irradiation as an Indoor Air Germicide 

**DOI:** 10.3390/ijerph110303271

**Published:** 2014-03-20

**Authors:** Thanh-Dong Pham, Byeong-Kyu Lee

**Affiliations:** Department of Civil and Environmental Engineering, University of Ulsan, Daehakro 93, Namgu, Ulsan 680-749, Korea; E-Mail: thanhdongpham080808@gmail.com

**Keywords:** germicide feasibility, Staph, silver doping, TiO_2_ coating, plasmon resonance

## Abstract

This study investigated the feasibility of using Ag-TiO_2 _photocatalyst supported on glass fiber (Ag-TiO_2_/GF) prepared by a sol-gel method as an indoor air germicide. An experimental model was designed to investigate the bacterial disinfection efficiency of *Staphylococcus* (Staph), the most popular bacterium in hospitals in Korea, by the Ag-TiO_2_/GF photocatalyst. The silver content in Ag/TiO_2_ was altered from 1 to 10% to investigate the optimal ratio of Ag doped on TiO_2_/glass fiber (TiO_2_/GF) for photocatalytic disinfection of Staph. This study confirmed that Ag in Ag-TiO_2_/GF could work as an electron sink or donor to increase photocatalytic activity and promote the charge separation of electron-hole pairs generated from TiO_2_ after photon absorption. Ag also acts as an intermediate agent for the transfer of photo-generated electrons from the valence band of TiO_2_ to an acceptor (O_2_ gas) to promote photo-oxidation processes. The photocatalytic disinfection activity of Ag-TiO_2_/GF under visible light increased with the increase in silver content up to 7.5% and then slightly decreased with further increasing silver content. The highest disinfection efficiency and disinfection capacity of Staph using 7.5% Ag-TiO_2_/GF were 75.23% and 20 (CFU∙s^−1^∙cm^−^^2^) respectively. The medium level of humidity of 60% ± 5% showed better photocatalytic disinfection than the lower (40% ± 5%) or higher (80% ± 5%) levels.

## 1. Introduction

The World Health Organization (WHO) has stated that air pollution is the sixth-leading cause of death globally, inducing over 2.4 million premature deaths per year worldwide in 2002, 1.5 million of which were attributable to indoor air pollution [1]. Exposure to bioaerosols can be associated with a wide range of adverse health effects including infectious diseases, acute toxic effects, allergies and cancer [2]. A bioaerosol is usually defined as a suspension of airborne particles that contain living organisms or is released from living organisms [3]. Bacteria, fungi, viruses, high molecular weight (HMW) allergens, bacterial endotoxins and mycotoxins are the main components of bioaerosols [2]. Among them, pathogenic bacteria can spread easily and rapidly in an aerosol, causing serious diseases, such as anthrax, tuberculosis, pneumonia, syphilis, gastroenteritis, *etc.* [4]. High efficiency particulate air (HEPA) filters are widely used in air conditioning systems to remove airborne particulates and microorganisms. However, filter application methods only transfer the microorganisms to another phase rather than eliminating them. In addition, air filters may themselves become a source of microbes if those retained microorganisms proliferate and are re-entrained into the filtered air [5]. Recently, the integration of photocatalysts into air purifiers has become more popular for dealing with the problems or drawbacks associated with the air filtration of bioaerosols [5,6,7,8,9].

Over the past decades titanium dioxide (TiO_2_) has been one of the most popular and successful photocatalysts for the remediation of contaminated water and air due to its exceptional optical and electronic properties, chemical stability, non-toxicity and low cost [10,11,12]. Matsunaga *et al.* first demonstrated the use of TiO_2_ as a biocide in 1985 [13]; since, there have been numerous studies on biological disinfection including the destruction of bacterial species [14,15,16,17,18], viruses [13,19], algae [20] and fungi [13,21]. The mechanisms of photocatalytic disinfection have been reported in various studies [22,23,24,25]. To be used as a photocatalyst, however, TiO_2_ should be irradiated with ultraviolet (UV) radiation. As energy that is higher than the band gap energy between the valence and the conduction bands is provided to the TiO_2_ catalyst, an electron in the valence band could be excited to jump to the conduction band, leaving a hole behind. The hole in the valence band reacts with surrounding water molecules or moisture to produce hydroxyl radicals (∙OH), while the electrons in the conduction band react with molecular oxygen to produce superoxide radical anions (∙O_2_^−^). These oxy radical species participate in oxidation reactions to attack vital organic components of microorganisms such as cell membranes, RNA, DNA, proteins and lipids, resulting in the death of microorganisms. Because of the wide band gap of TiO_2_ (3.2 eV) between the valence and conduction bands, however, visible light cannot be used as a light resource to excite electrons to initiate the photocatalytic disinfection of microorganisms. Thus, enhancing the photocatalytic activity of TiO_2_ to expand its application under visible light is one of the most important tasks for the practical application of TiO_2_ in the future.

A large number of studies have been carried out to improve the photocatalytic activity of TiO_2_ [26,27,28,29,30]. Compared with pure TiO_2_, modified TiO_2_ techniques have many advantages in light absorption, charge recombination dynamics, interfacial charge transfer kinetics, surface structure and charge and adsorption of substrates, resulting in photocatalytic enhancement. Ag is of particular interest as a doping agent to enhance TiO_2_ photocatalytic activity due to its ability to act as both an electron sink and redox catalyst [31]. While acting as an electron sink to attract photoelectrons, Ag promotes the charge separation of electron-hole pairs from TiO_2_ after photon absorption, resulting in prevention of the fast recombination of photogenerated charge carriers [32]. The plasmon resonance in metallic silver (Ag particles) can also enhance the electric fields, facilitating electron–hole production [32]. Ag is one of the most promising metals for improving the photocatalytic disinfection property of TiO_2_ due to its strong and broad bactericides [33]. It is difficult to separate and regenerate photocatalytic powders from purification systems after being used as photocatalysts for water or air purification [34]. To overcome the disadvantages of photocatalysis powders, various methods have been developed, including a technique for coating TiO_2_ on many substrates [35]. In this study, glass fiber was used as a substrate for Ag-TiO_2_ coating to prepare photocatalyst Ag-TiO_2_/GF for photocatalytic disinfection of bioaerosols under visible light. 

There have been few studies applying photocatalytic disinfection in the bioaerosol field [36]. Humidity plays a vital role regarding hydroxyl radical production in TiO_2_ photocatalytic systems. However, the effect of humidity on photocatalytic disinfection has not been clearly reported yet. The main purpose of this study is to investigate optimum loading ratios of photocatalyst Ag-TiO_2_/GF to improve photocatalytic efficiencies for bioaerosol disinfection under visible light, which has many advantages compared to UV irradiation in terms of energy consumption and application feasibility. This study also aims to investigate the effect of humidity on photocatalytic disinfection of Ag-TiO_2_/GF for bioaerosol removal.

## 2. Materials and Methods

### 2.1. Catalyst Preparation

A clear solution of titania was prepared from the dissolution of tetraisopropyl orthotitanate (TIOT) and nitric acid (HNO_3_) in distilled water with stirring for 8 h. The clear solution was used to prepare a colloidal solution of titania by holding the temperature at 60 °C for 8 h. The glass fiber with area 240 cm^2^ was immersed in the colloidal solution to prepare the TiO_2_-coated glass fiber, which was dried at 60 °C for 6 h after being removed from the colloidal solution. The designated thickness of the TiO_2_ layer was produced using two repeated processes and was obtained as the weight fraction of TiO_2_ in the TiO_2_/GF at 3%. A solution of 0.05 M AgNO_3_ was dropped on the 3% TiO_2_/GF to prepare Ag-TiO_2_/GF photocatalyst. These Ag-TiO_2_/GF photocatalysts with weight fractions of 1, 2.5, 5, 7.5 and 10% Ag in the Ag/TiO_2_ ratio were prepared by adjusting the volume addition of the AgNO_3_ solution. Before being used for the bacterial disinfection process all the catalyst materials were calcined at 200 °C for 2 h.

### 2.2. Catalyst Characterization

A Hitachi S-4700 scanning electron microscope (SEM) was used to determine the surface morphologies of the Ag-TiO_2_/GF photocatalytic materials. X-ray diffraction (XRD) patterns were obtained using a Bruker AXN model with a Cu-Kα radiation (*λ* = 1.5418 Å) source operated at a scan rate of 0.02 s^−1^ over a 2θ range of 10–80°. A Varian Cary 500 was used to record the UV-visible diffuse reflectance spectra (DRS) of photocatalysis materials. X-ray photoelectron spectroscopy (XPS) measurements were performed using a Thermo Fisher K-alpha model to determine the chemical composition of Ag-TiO_2_/GF photocatalysts.

### 2.3. Disinfection Experiment

**Figure 1:**
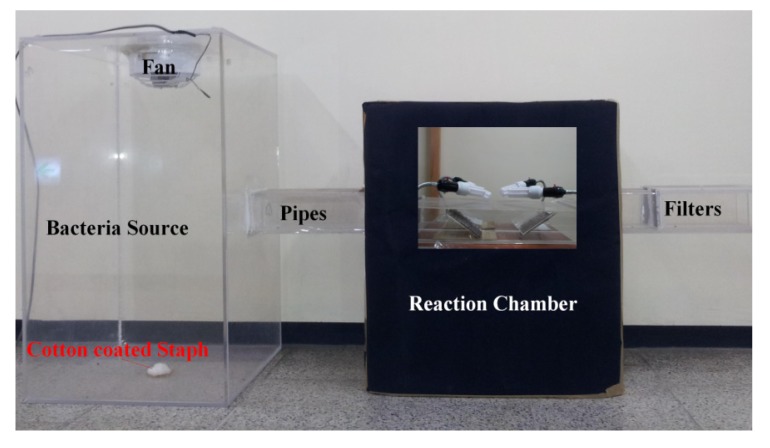
Schematic model of the bacterial photocatalytic disinfection system.

For bacterial disinfection efficiency tests, this study used *Staphylococcus* obtained from an environmental bioengineering laboratory, University of Ulsan, Korea. For the cultivation of Staph, we applied a Luria-Bertani medium containing bacto tryptone (10 g/L), bacto yeast extract (5 g/L) and NaCl (5 g/L). Bacteria were incubated at 37 °C for 12 h on a rotary shaker and then the cultures of bacteria were centrifuged at 5,000 rpm for 10 min in a bench centrifuge and the cells were coated on cotton. The Staph cotton was placed on the center of the bacterial source cask bottom to use as a Staph source for each disinfection experiment (Figure 1). Tris-buffered saline (TBS) solution, a mixture of 0.15 M NaCl and 0.05 M Tris-HCl at pH 7.5, was used to transfer Staph from the filter to a test solution. Then, the test solution was diluted with saline and spread onto an agar plate. The agar plate was kept at 37 °C for 8 h, and then the bacteria colonies were counted. The number of bacteria in the original filter was used as a basis to calculate the dilution factor.

An experimental model was designed with a two-parallel-pipes system for bioaerosol photocatalytic disinfection (Figure 1). The amount of Staph and humidity inside the model could be easily adjusted before each photocatalytic disinfection experiment. More details regarding the model design and safe conditions were presented in a sister paper [37]. The constant temperature inside the experimental model system was 25 °C during the disinfection process. Four 20 W white-light lamps were used to obtain visible light to initiate the electron transfer from the valence band to the conduction band of TiO_2_. The effective power density was 0.025 W/cm^2^. The reaction time of each photocatalytic disinfection experiments was 1 h. 

In this study, the photocatalytic disinfection efficiencies of Staph using 1, 2.5, 5, 7.5 and 10% Ag-TiO_2_/GF photocatalysts were compared in order to investigate the optimal content of Ag doped on the TiO_2_/GF. The identified optimal photocatalyst was used for photocatalytic disinfection of Staph under different relative humidity (RH) conditions (40% ± 5%, 60% ± 5%, 80% ± 5%) to determine the effect of humidity on photocatalytic disinfection. This study also analyzed the effects of different input amounts of Staph to determine disinfection capacity of the identified optimal photocatalyst while keeping the same reaction conditions of time, temperature and relative humidity. The Staph disinfection efficiency was calculated by Equation (1):

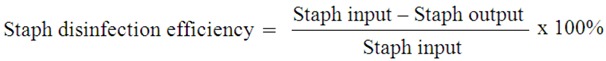
(1)

The Staph disinfection capacity was calculated using Equation (2) with a disinfection time (t) of 3,600 seconds and a photocatalytic area (S) of 240 cm^2^:

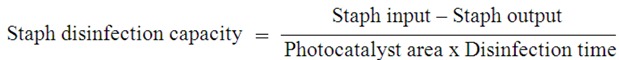
(2)

## 3. Results and Discussion

### 3.1. Photocatalyst Characterization

#### 3.1.1. SEM Observation

**Figure 2:**
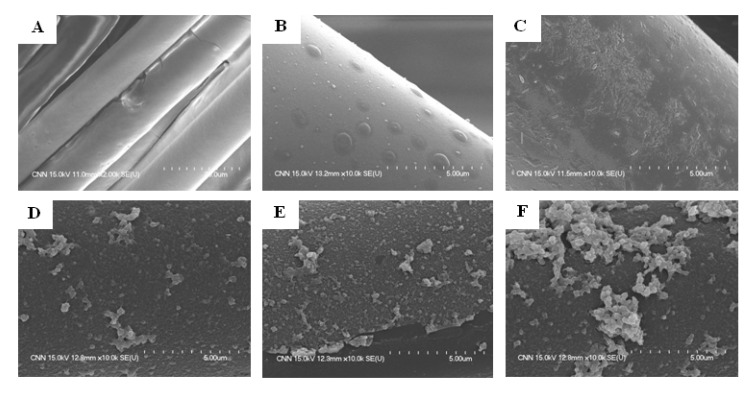
SEM photographs of 0 (**A**), 1 (**B**), 2.5 (**C**), 5 (**D**), 7.5 (**E**) and 10% (**F**) Ag-TiO_2_/GF.

Figure 2 shows the SEM images of 0, 1, 2.5, 5, 7.5 and 10% Ag-TiO_2_/GF photocatalysts. Glass fiber was used as the substrate for TiO_2_ deposition. TiO_2_ in the TiO_2_/GF was flat-deposited on the surface of the glass fiber (Figure 2A). 

Figure 2 shows that some Ag particles were dispersedly deposited on the surface of the TiO_2_ layer while other Ag particles slightly overlapped on the TiO_2_ layer. The Ag particle size simultaneously increased with increasing Ag content. Ag clustered into big particles on the TiO_2_ layer at high Ag-doping. Too much Ag gathering into larger particles of silver could create negative effects, including light eclipsed by the large silver particles with TiO_2_ and the uneven distribution of silver on the TiO_2_ layer, which could result in the decrease of photocatalytic activity. At lower silver doping (1% and 2.5% Ag-TiO_2_/GF), the doped amount of silver resulted in few interactions between silver and TiO_2_ and was therefore not enough to enhance the photocatalytic activity of TiO_2_. The optimal ratio of silver doped on TiO_2_ was determined based on characterization methods such as XRD, UV-VIS and XPS as well as Staph photocatalytic disinfection efficiency and capacity.

#### 3.1.2. XRD Analysis

**Figure 3:**
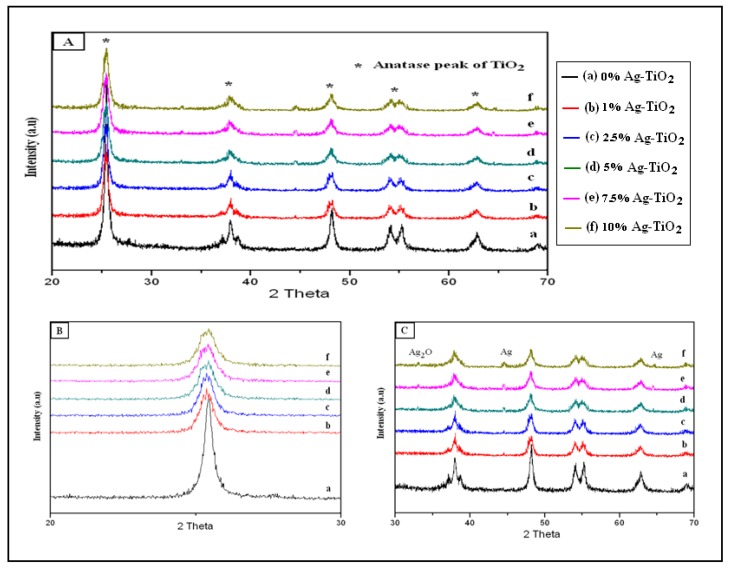
(**A**) XRD patterns of the TiO_2_ and 1, 2.5, 5, 7.5, and 10% Ag-TiO_2_; (**B**) XRD patterns of the TiO_2_ and 1, 2.5, 5, 7.5, and 10% Ag-TiO_2 _in the 2θ range of 20–30°; (**C**) XRD patterns of the TiO_2_ and 1, 2.5, 5, 7.5, and 10% Ag-TiO_2 _in the 2θ range of 30–70°.

In the Ag-TiO_2_/GF catalyst system, the intensity of the X-ray diffraction peaks of the TiO_2_ coated on the glass fiber was too weak for phase analysis due to the limited availability of TiO_2 _particles coated on the glass fiber. To determine the crystalline phase of TiO_2_ and Ag deposited on TiO_2_ using XRD peak analysis, TiO_2_ powder and 1, 2.5, 5, 7.5 and 10% Ag doped TiO_2_ powder were prepared from the colloidal solution of TiO_2_ without the immersion of the glass fiber. Figure 3A shows that the anatase phase of TiO_2_ was identified in XRD patterns of TiO_2_ and 1, 2.5, 5, 7.5 and 10% Ag doped TiO_2_. 

To analyze changes in X-ray peak intensity of the anatase peak of TiO_2_ as a function of the amount of doped Ag, this study magnified the XRD patterns of TiO_2_ and 1, 2.5, 5, 7.5 and 10% Ag doped TiO_2_ in the 2θ range 20–30°(Figure 3B). In the XRD patterns, the diffraction angles shown at 26° in 2θ corresponds to the anatase (101) peak of TiO_2_. The anatase peak intensity and shape of the TiO_2_ in the Ag doped TiO_2_ samples decreased and broadened, respectively, with increasing Ag doping, compared to the pure TiO_2 _sample. This is possibly due to the fact that Ag^+^ ions are deposited onto the surface of TiO_2_ particles. In addition, a slight shift of the anatase (101) peak to a smaller diffraction angle was recorded for the Ag doped TiO_2_ samples. This is because Ag^+^ entered the crystal structure of TiO_2_ and consequently induced distortion in the crystal lattice of TiO_2_. Liu *et al.* reported that silver could defect the lattice of TiO_2_ because the substitution of silver ions into the lattice sites of TiO_2_ induced O vacancies or deficiencies of Ti^4+^ [38]. In the TiO_2_ lattice, the radius of Ti^4+^ ion is 68 Å. However, the ionic radius of Ag^+^ ions is 126 Å. As such, substitution or replacement of Ag^+^ ions for Ti^4+^ ions in TiO_2_ lattice requires high energy [39] and therefore only a small amount of Ag^+^ ions would enter the TiO_2_ lattice and distort the lattice structure. The more Ag doping onto TiO_2,_ the more distortion there is in the TiO_2_ lattice, which results in greater peak broadening, increased shifting to lower angles and greater decreases in anatase peak intensity.

Figure 3C shows XRD patterns of TiO_2_ and 1, 2.5, 5, 7.5, and 10% Ag doped TiO_2 _in the 2θ range of 30–70°. An Ag metallic peak was observed at 44.4° in Ag doped TiO_2_ samples. When the Ag content increased up to 5%, one more weak Ag metallic peak occurred at 64.5°. A weak Ag_2_O peak was also notably observed at 33.1° when the Ag content in Ag doped TiO_2_ samples increased from 5% to 10%. 

The occurrence of a small amount of silver oxide (Ag_2_O) in the samples suggested that at the calcination temperature around 200 °C, thermal decomposition of AgNO_3_ into Ag_2_O was observed in the main process of decomposition of AgNO_3_ into Ag metallic via Ag_2_O. Gao *et al.* reported that the thermal decomposition of Ag_2_O phases to Ag and oxygen atoms transpires at T_a_ ≥ 200 °C [40]. Even though, the Ag-O bonding in Ag_2_O is much weaker than Ag-Ag bonding in Ag lattice at 200°C, there is a possibility of small amounts of Ag_2_O remaining from the decomposition of AgNO_3_ [41]. The Ag and Ag_2_O produced from thermal decomposition of AgNO_3_ is explained by the following reaction scheme:

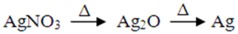
(3)

#### 3.1.3. UV-Visible Spectra

**Figure 4:**
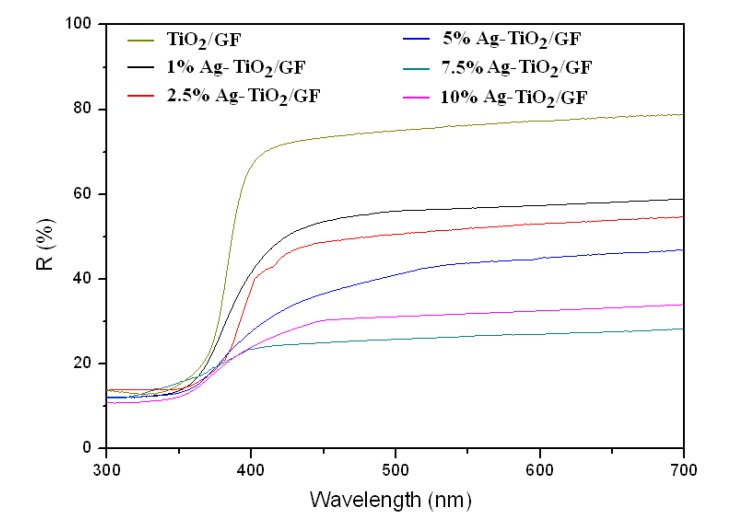
UV-Visible diffuse reflectance spectra of the TiO_2_/GF and 1, 2.5, 5, 7.5 and 10% Ag-TiO_2_/GF.

Figure 4 shows the optical absorption properties of the TiO_2_/GF and 1, 2.5, 5, 7.5 and 10% Ag-TiO_2_/GF. The Ag-TiO_2_/GF samples exhibited an obvious red-shift of absorbance edge and a significant enhancement of light absorption in the region of 300–800 nm. Since the Fermi level of Ag is lower than that of TiO_2_, Ag on the surface of the TiO_2_ layer acts as an intermediate agent for the transfer of photo-generated electrons from the valence band of TiO_2_ to an acceptor (O_2_ gas) [42]. Therefore, Ag can enhance the photogenerated electron-hole pair separation of TiO_2_ and inhibit their recombination resulting in increased visible light absorption. 

The visible light adsorption of Ag-TiO_2_/GF increased as the Ag content increased up to 7.5% and then began to drop when Ag content was increased up to 10%. The visible adsorption of Ag-TiO_2_/GF proportionally increased with increasing Ag content. The plasmon resonance of Ag particles dispersing into the TiO_2_ layer increased with increasing Ag content. The increase in the plasmon resonance can enhance electric fields, which can facilitate electron-hole production resulting in increased visible adsorption [43]. However, when the Ag content is too high, e.g., 10% in Ag-TiO_2_/GF, the plasmon resonance can inhibit the electron-hole separation of TiO_2_, resulting in decreased visible light adsorption. At the high silver loading, the size of the silver clusters became too large, thus blocking light from reaching the TiO_2_ surface. Therefore, the chances of visible light reaching the TiO_2_ layer decreased resulting in less visible light for adsorption to the photocatalyst [44]. 

#### 3.1.4. XPS Studies

**Figure 5:**
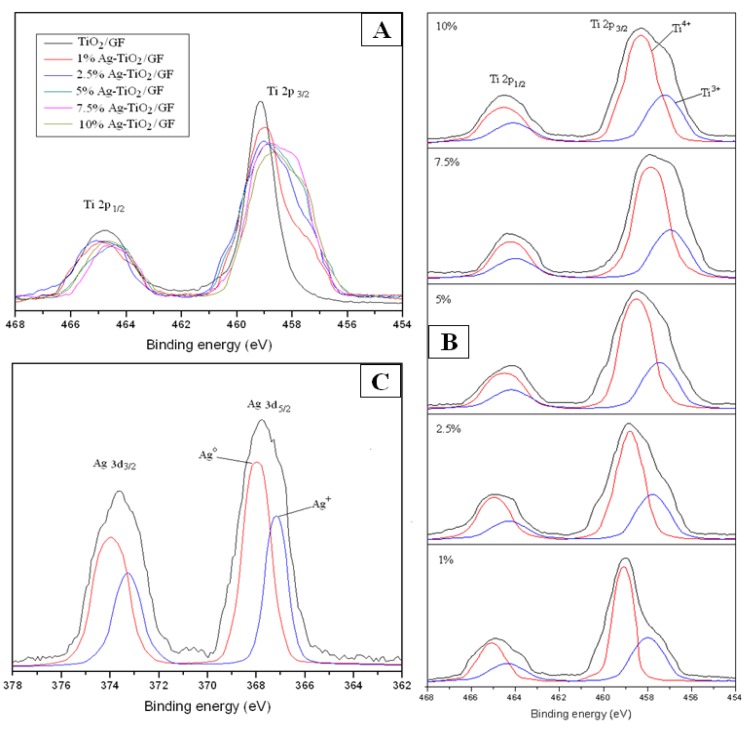
(**A**) XPS spectra of Ti 2p of TiO_2_/GF and 1, 2.5, 5, 7.5 and 10% Ag-TiO_2_/GF, (**B**) High resolution XPS spectra of Ti 2p in X% Ag-TiO_2_/GF, (**C**) XPS of Ag 3d in 7.5% Ag-TiO_2_/GF.

Figure 5A shows the high-resolution XPS spectra of titanium (Ti) in TiO_2_/GF and 1, 2.5, 5, 7.5 and 10% Ag-TiO_2_/GF. The Ti 2p_3/2 _peak and the Ti 2p_1/2 _peak of TiO_2_ in the TiO_2_/GF samples appeared at binding energies of 459.18 eV and 465.18 eV, respectively. The doublet splitting energy of Ti 2p peaks was 6.0 eV, which was attributed to Ti^4+^ in the lattice of titanium. The narrow and sharp peaks of Ti 2p in XPS spectra of TiO_2_/GF indicated that all Ti ions in TiO_2_ existed in the Ti^4+^ state [45]. Figure 5A shows peak broadening and a shift into lower binding energy of the Ti 2p peaks of TiO_2_ in Ag-TiO_2_/GF materials. The peak broadening of Ti 2p_1/2 _and Ti 2p_3/2 _in Ag-TiO_2_/GF implies that the ion state of titanium in Ag-TiO_2_/GF was not only Ti^4+^. Gaussian multipeak shapes were applied to fit Ti 2p peaks of TiO_2_ in the Ag-TiO_2_/GF (Figure 5B). XPS spectra of the Ag-TiO_2_/GF photocatalysis showed the presence of two peaks matching Ti^3+ ^and Ti^4+^ [46]. The Ti^3+^ might have formed during the calcination process (oxidation of tetra isopropyl ortho titanate (TIOT)) to prepare the TiO_2_ layer with the assistance of Ag or Ag^+^ ion. Ag or Ag^+^ ion can extract an electron from isopropyl radical (^−^C_3_H_7_) during the calcination process, and then the extracted electron transfers to Ti^4+^ to form Ti^3+^ [47,48]. These reactions are summarized as follows:

e^−^ + Ag → Ag^−^ (4)

Ag^−^ + Ti^4+^ → Ag + Ti^3+^ (5)

Ti^4+^ and O^2−^ from TIOT during the calcination process would experience rearrangement to produce TiO_2_. The rearrangement can be disturbed by Ag^+^ resulting in changes in the TiO_2_ lattice [38,49]. When an Ag ion replaces Ti^4+ ^ion in a lattice site, it would induce O vacancies or deficiencies of Ti^4+ ^in the TiO_2_ lattice, resulting in some changes of Ti^4+^ ions into Ti^3+ ^ions [38,39]. As explained in the XRD analysis section, the radius of Ag^+^ (126 Å) is much larger than that of Ti^4+^ (68 Å). The replacement of Ag^+^ into the TiO_2_ lattice requires energy and thus only a small fraction of Ti^4+ ^is replaced by Ag^+^ leading to only small amounts of Ti^3+^. This indicates that the fraction of Ti^4+ ^in TiO_2_ lattice is dominant compared to Ti^3+^ and thus the peak intensity of Ti^4+ ^is much higher than that of Ti^3+ ^(Figure 5B). The Ti^4+^ and Ti^3+^ peak positions also shifted into lower binding energy fields as the doped amount of silver in the TiO_2_ lattice increased (Figure 5B). The plasmon resonance of silver could expand the titanium ion radius resulting in electron movement far from the titanium nuclei; therefore, the peaks of Ti 2p shifted into a lower binding energy [50]. The increase of the plasmon resonance of silver was proportional to the increase in silver content. The Ag^+ ^present on the outside of the titanium ions can contribute to the expansion of the electron cloud around titanium nuclei thereby increasing the titanium radius and the further release of electrons. This phenomenon is observed through Ti 2p peak shifts into lower binding energies (Figure 5B). The occurrence of Ti^3+^, which contains one more electron than Ti^4+^, might promote electron generation from TiO_2_ when the Ag-TiO_2_ photocatalysts are excited by irradiation. Ag in TiO_2_ lattice can affect expansion of the titanium ion radius, which is demonstrated by the Ti^3+^ and Ti^4+^ peak shifts into lower binding energies (Figure 5B). Hence, the easily developed charge separation of electron-hole pairs from TiO_2_ could require relatively lower energy to reach excitation for photoreaction. This is the reason that photocatalytic activity can occur even under visible light. If the silver content is too high, *i.e.* 10%, silver ions can make more complexes around titanium. If silver ions exist at the opposite site of the titanium molecules, an electron cloud can develop opposite, thus withdrawing forces onto titanium. This kind of plasmon resonance can decrease the expansion power of the titanium radius. Therefore, the peaks of Ti 2p in 10% Ag-TiO_2_/GF shift slightly into higher binding energy fields compared to 7.5% Ag-TiO_2_/GF.

Figure 5C shows the XPS result of Ag 3d of 7.5% Ag-TiO_2_/GF. The Gauss multipeak shapes showed that the Ag 3d XPS peak was decomposed into two peaks for Ag_2_O and Ag metallic. The simultaneous occurrence of Ag and Ag_2_O in Ag-TiO_2_ was also reported by Zhang *et al.* [45]. At the calcination temperature around 200 °C, AgNO_3_ decomposed into Ag as well as Ag_2_O due to the reciprocal conversion between Ag^0^ and Ag^+^. This conversion is described by the following reaction:

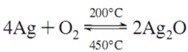
(6)

Hence, the Ag-TiO_2_/GF essentially includes Ag and Ag_2_O, which are deposited on the TiO_2_/GF. However, both Ag metallic and Ag_2_O are able to enhance photocatalytic activity of TiO_2_ via sensitization by their plasmon resonance [45,50].

### 3.2. Disinfection Results

#### 3.2.1 Optimum Ag Doping

Table 1 shows the Staph disinfection results at different Ag loading in Ag-TiO_2_/GF under visible light. The Ag-TiO_2_/GF photocatalyst showed very high disinfection efficiencies of Staph, even under visible light. This great improvement of Staph disinfection by the photocatalyst Ag-TiO_2_/GF is a result of the Ag doping. Ag enhanced the electron-hole separation of TiO_2_ resulting in improved photocatalytic activity under visible light. The electron-hole pairs could be easily generated when the Ag-TiO_2_/GF was irradiated even by visible light, and then the generated electron-hole pairs reacted with water and molecular oxygen absorbed on the photocatalysis surface to form hydroxyl radicals (∙OH) and superoxide radical anions (∙O_2_^−^) [41]. These oxy radicals participate in the oxidation of organic components of Staph resulting in bacterial death [51]. Ag also acted as an intermediate agent for the transfer of photo-generated electrons from the valence band of TiO_2_ to an acceptor (O_2_ gas) to produce superoxide radicals. The Ag-TiO_2_/GF system mainly undergoes the following reactions:

TiO_2_ + hν → e^−^ + h^+^ (7)

h^+^ + H_2_O → H^+^ + ∙OH (8)

e^−^ + Ag → Ag^−^ (9)

Ag^−^ + O_2_ → Ag + O_2_^−^ (10)

O_2_^−^ + e^−^ + H^+^ → H_2_O_2_ (11)

H_2_O_2_ → ∙OH (12)

∙OH + organic compounds (DNA, RNA, lipid) → CO_2_ + H_2_O (13)

**Table 1:** Staph disinfection results of different Ag loading in Ag-TiO_2_/GF.

Ag Doping in TiO_2_ (%)	0	1	2.5	5	7.5	10
Staph input (CFU)	2.14E + 07	2.11E + 07	2.08E + 07	2.03E + 07	2.16E + 07	2.06E + 07
Staph output (CFU)	2.08E + 07	9.75E + 06	7.90E + 06	6.50E + 06	5.35E + 06	5.95E + 06
Disinfection efficiency (%)	2.80	53.79	62.02	67.98	75.23	71.12

Table 1 shows the disinfection efficiency of Staph by the Ag-TiO_2_/GF photocatalysts with different amounts of silver doping. The disinfection efficiency gradually increased with increasing Ag content in Ag-TiO_2_/GF photocatalyst. The highest disinfection efficiency was 75.23% with Ag-TiO_2_/GF at 7.5% Ag loading. When the silver content was increased up to 10%, the disinfection efficiency began to drop. This result coincided with the preliminary conclusion inferred from the results in the SEM observation, UV-Vis spectra and XPS studies. When the silver content is increased too much, Ag clustered into larger size and non-uniform particles on the TiO_2_ surface. This leaded to limit the amount of light, H_2_O and O_2_ that were able to reach the TiO_2_ layer, resulting in decreased hydroxyl radical generation, which is a main factor for Staph disinfection [52]. The shrinkage of the titanium ion radius was evident in the peak shift to the high binding energy of Ti 2p in the photocatalytic system with 10% Ag-TiO_2_/GF. This result indicates that the 10% Ag-TiO_2_/GF has relative difficultly in generating electron-hole pairs compared to the 7.5% Ag-TiO_2_/GF. This also means the photocatalytic disinfection ability of Staph of the 10% Ag-TiO_2_/GF will be lower than that of the 7.5% Ag-TiO_2_/GF.

#### 3.2.2. Humidity Effects

The effects of relative humidity on photocatalytic disinfection efficiency of Staph are shown in Table 2. Among the three tested relative humidity (RH) conditions, the highest disinfection efficiency of 75.23% was observed at RH 60% ± 5%. Under the dry humidity condition (40% ± 5% RH), the disinfection efficiency was 52.93%. The decrease in disinfection efficiency under the dry humidity condition was due to the lack of available H_2_O molecules for hydroxyl radical generation by photocatalytic reaction. Hydroxyl radical generation or availability is a major factor affecting bacterial disinfection [52]. Therefore, the lack of hydroxyl radical would greatly decrease disinfection efficiency. At the humid condition (80% ± 5% RH), the disinfection efficiency of Staph was 65.95%. This was higher than the disinfection efficiency in the dry condition, but lower than that of the medium humidity condition (60% ± 5%). According to the explanation of this phenomenon by Li *et al.*, high humidity could induce reactivation of organisms, or water may occupy most of the TiO_2_ sites resulting in fewer sites available for disinfection or adsorption of microorganisms [53]. At a certain humidity level, the presence of water vapor could promote hydroxyl radical formation, however, the radical formation would not always increase with increasing water vapor. Sometimes, the radicals can even decrease due to the occupation of the adsorption site on the TiO_2_ surface, resulting in decreased disinfection efficiency at high humidity conditions [53]. Peccia *et al.* reported that biopolymers interior to the cell or protein structure could be changed at a certain level of relative humidity [54]. The protein structure change could affect the DNA repair enzyme, including cell wall characteristics, and hence could protect the microorganism from oxidation by hydroxyl radicals. Therefore, microorganisms can better survive when humidity is high, resulting in a slight decrease in the disinfection efficiency of Staph.

**Table 2:** Effects of relative humidity on photocatalytic disinfection efficiency of Staph.

Relative Humidity (%)	40 ± 5	60 ± 5	80 ± 5
Staph input (CFU)	2.05E + 07	2.16E + 07	2.10E + 07
Staph output (CFU)	9.65E + 06	5.35E + 06	7.15E + 06
Disinfection efficiency (%)	52.93	75.23	65.95

#### 3.2.3. Staph Disinfection Capacity of Optimal Photocatalysis

**Figure 6:**
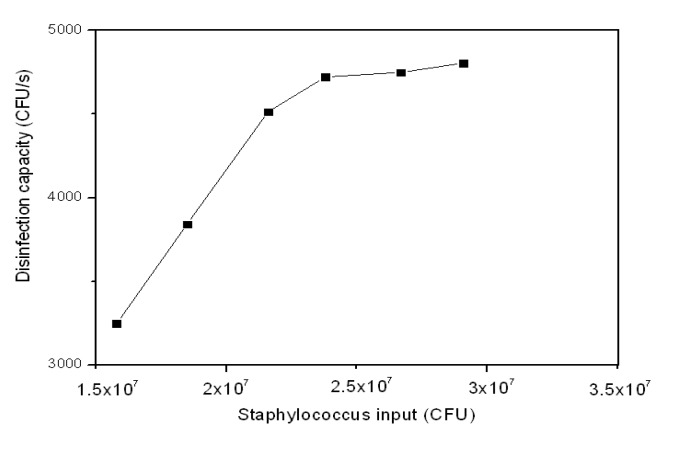
Disinfection capacity of Staph by 7.5% Ag-TiO_2_/GF photocatalyst at different Staph inputs.

Table 3 shows the disinfection capacity of Staph by 7.5% Ag-TiO_2_/GF photocatalyst under visible light when the Staph input amount ranged from 1.58 × 10^7^ to 2.91 × 10^7^ (CFU). The disinfection capacity was calculated in CFU∙s^−1^ as well as CFU.s^−1^.cm^−2^. The disinfection capacity of the photocatalysis increased as the Staph input increased. The disinfection capacity almost reached the limit when Staph input was higher than 2.38 × 10^7^ (CFU). The disinfection capacity increased from 3,247 (CFU∙s^−1^) to 4,806 (CFU∙s^−1^) when the Staph input increased from 1.58 × 10^7^ to 2.91 × 10^7^ (CFU). The disinfection capacity seemed to be stable around 4,750 (CFU∙s^−1^). Thus, there was only a slight increase in the disinfection efficiency accompanying the substantial increase in Staph remaining without disinfection even with further increased in Staph input. This is due to the limited number of available hydroxyl radicals generated per second by the photocatalytic activity resulting in an increase in untreated Staph, *i.e.*, those not disinfected by the photocatalytic system. Although there is slight increase in disinfection capacity above 4,750 (CFU∙s^−1^) the disinfection capacity in CFU∙s^−1^∙cm^−^^2^ did not increase further after reaching 20 (CFU∙s^−1^∙cm^−^^2^) (Figure 6).

**Table 3:** Disinfection capacity of 7.5% Ag-TiO_2_/GF with different Staph inputs.

Staph Input (CFU)	Staph Output (CFU)	Disinfection Capacity		Staph Remained (CFU·s^−1^)
		(CFU·s^−1^)	(CFU·s^−1^·cm^−2^)	
1.58E + 07	4.11E + 06	3,247	14	1,142
1.85E + 07	4.67E + 06	3,842	16	1,297
2.16E + 07	5.35E + 06	4,514	19	1,486
2.38E + 07	6.80E + 06	4,722	20	1,889
2.67E + 07	9.60E +06	4,750	20	2,667
2.91E + 07	1.18E + 07	4,806	20	3,278

**Figure 7:**
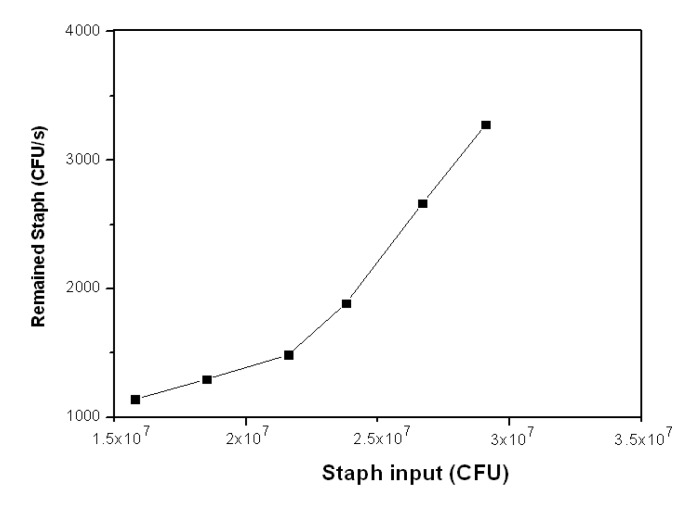
Remaining Staph at different input amounts.

Figure 7 shows the Staph which was still alive and on the filter after the disinfection experiments. When the Staph input changed from 1.58 × 10^7^ to 2.16 × 10^7^ (CFU), the Staph remaining on the filter after the disinfection experiments increased from 1,142 (CFU∙s^−1^) to 1,486 (CFU∙s^−1^), which is only a small increase. 

This slight change suggests that there was a small amount of Staph input that was not affected by hydroxyl radicals available on the surface of the photocatalyst. However, there was great increase in the Staph that was still alive and on the filters after the photocatalytic disinfection as the Staph input increased from 2.16 × 10^7^ (CFU) to 2.91 × 10^7^ 16 × 10^7^ (CFU). This means that a large amount of Staph was just passing through the photocatalytic pipe without being disinfected even with hydroxyl radicals generated from the surface of the photocatalyst. This also indicates there is a limited number of generated hydroxyl radicals available for disinfection under the given photocatalytic system.

## 4. Conclusions

This study investigated the chemical mechanism of photocatalytic disinfection of Staph using Ag-TiO_2_/GF under visible light. Silver doping influenced the microstructure of the photocatalyst. Increased silver loading produced a greater number of radicals, which can increase Staph disinfection via increased electron-hole separation and the formation of more intermediate agents to facilitate the transfer of electrons from valence band to acceptor (O_2_ gas). Loading too much silver decreased the photocatalytic disinfection efficiency because too much silver on the TiO_2_ layer blocked effective contact between TiO_2_ and H_2_O as well as between Staph and oxidative radicals.

Too dry and too humid environmental conditions both decreased the photocatalytic disinfection efficiency because of the lack of available H_2_O to produce hydroxyl radicals in the dry condition (40% ± 5% RH) and because of the blockage of effective contact between Staph and radicals by too much H_2_O in the high humid condition (80% ± 5% RH). The identified optimum silver loading and humidity conditions for the disinfection of Staph were 7.5% Ag loading in the Ag-TiO_2_/GF photocatalyst and 60% ± 5% RH, respectively. The photocatalytic disinfection capacity of Staph under the optimum silver loading and humidity conditions was 4,750 (CFU∙s^−1^) or 20 (CFU∙s^−1^∙cm^−^^2^)
